# Laparoscopic approach to recurrence following multiple surgeries for external rectal prolapse: a case report

**DOI:** 10.1186/s40792-021-01154-2

**Published:** 2021-03-19

**Authors:** Kosuke Toda, Taro Aoyama, Kenjiro Hirai, Taisuke Uemura, Haruku Fujita, Asami Okabe, Hidenori Ohe, Tsuyoshi Tachibana, Akira Mitsuyoshi

**Affiliations:** Department of Surgery, Otsu City Hospital, 2-9-9 Motomiya, Otsu, Shiga 520-0804 Japan

**Keywords:** Rectal prolapse, Pelvic organ prolapse, Recurrence, Laparoscopic ventral mesh rectopexy, Laparoscopic sacrocolpopexy

## Abstract

**Introduction:**

The optimal procedure for recurrent external rectal prolapse remains unclear, particularly in laparoscopic approach. In addition, pelvic organ prolapse (POP) is sometimes concomitant with rectal prolapse. We present a case who underwent laparoscopic procedure for the recurrence of full-thickness external rectal prolapse coexisting POP.

**Case presentation:**

An 81-year-old parous female had a 10-cm full-thickness external rectal prolapse following the two operations: the first was perineal recto-sigmoidectomy and the second was laparoscopic posterior mesh rectopexy. Imaging study revealed that the recurrent rectal prolapse was concomitant with both cystocele and exposed vagina, what we call POP. We planned and successfully performed laparoscopic ventral mesh rectopexy (LVMR) with laparoscopic sacrocolpopexy (LSC) using self-cut meshes without any perioperative complication.

**Conclusion:**

This is the first report of LVMR and LSC for recurrent rectal prolapse with POP following the perineal recto-sigmoidectomy and laparoscopic posterior mesh rectopexy. Even for recurrent rectal prolapse with POP, our experience suggests that LVMR and LSC could be utilized.

## Introduction

Rectal prolapse is a common disease in elderly women. The surgical procedures for rectal prolapse are divided into two main approaches: abdominal or perineal. Recently, laparoscopic ventral mesh rectopexy (LVMR) for primary external rectal prolapse is becoming one of the major procedures [1, 2]. Pelvic organ prolapse (POP) is also a common disease in 50% of parous women [3]. The prevalence of POP involving both urinary incontinence and genital prolapse in women with rectal prolapse has been reported to be 31% [4]. Furthermore, the patients who were undergoing surgical repair for rectal prolapse with POP significantly had higher rate of recurrent rectal prolapse than those without POP [5].

Laparoscopic sacrocolpopexy (LSC) is recognized as the gold standard for POP [6]. Meanwhile, the optimal surgical procedure for recurrent rectal prolapse is still unclear. In addition, there are a few reports that described the technical point of laparoscopic procedure for the recurrent rectal prolapse. Here, we present a case who successfully underwent the laparoscopic procedure for recurrent rectal prolapse with POP following the perineal recto-sigmoidectomy and the laparoscopic posterior mesh rectopexy.

## Case presentation

**Fig. 1:**
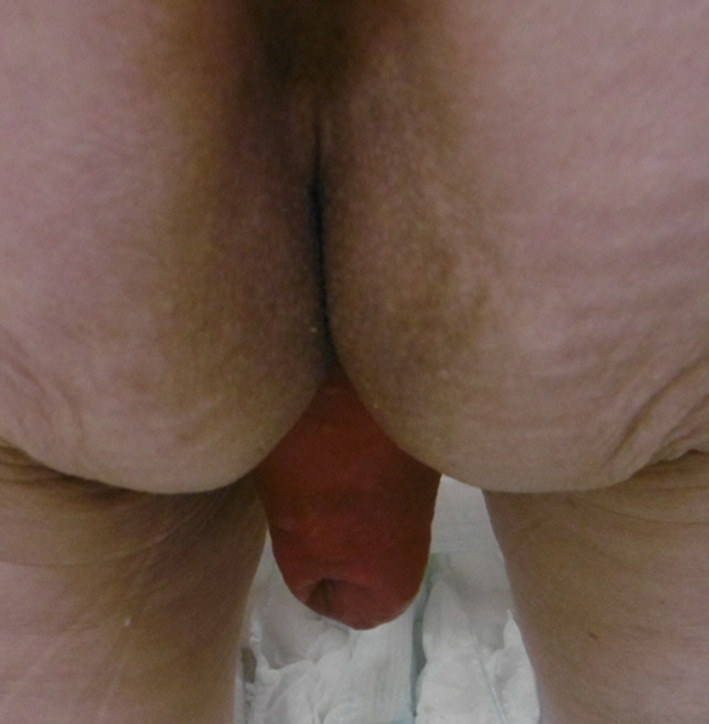
The recurrent rectal prolapse

**Fig. 2:**
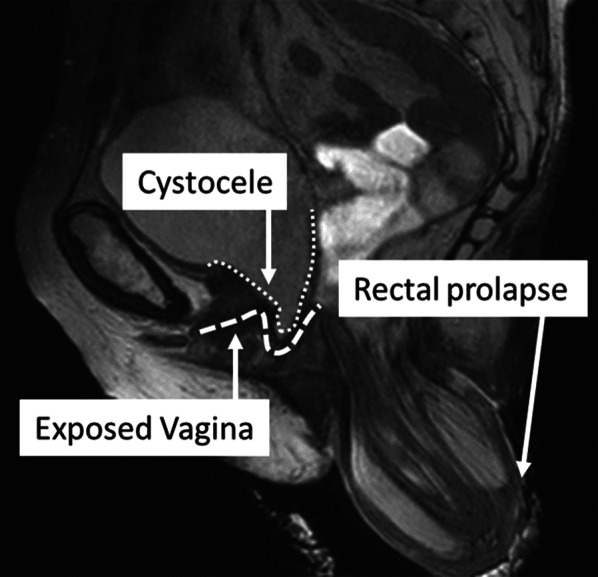
The magnetic resonance Image (MRI) revealed the cystocele and the exposed vagina with rectal prolapse

An 81-year-old parous female had multiple surgeries for rectal prolapse. Perineal recto-sigmoidectomy and laparoscopic posterior mesh rectopexy were performed 6 years and 3 years ago, respectively. According to the previous operation records, 15 cm rectosigmoid-colon was resected with levatorplasty on perineal recto-sigmoidectomy. Laparoscopic posterior mesh rectopexy was Wells procedure. She presented a 10-cm full-thickness external rectal prolapse (Fig. 1). As the magnetic resonance image (MRI) showed the cystocele and the exposed vagina with rectal prolapse, we diagnosed POP (Fig. 2). Accordingly, we planned to perform LVMR with LSC for recurrent rectal prolapse with POP.

## Surgical technique

**Fig. 3:**
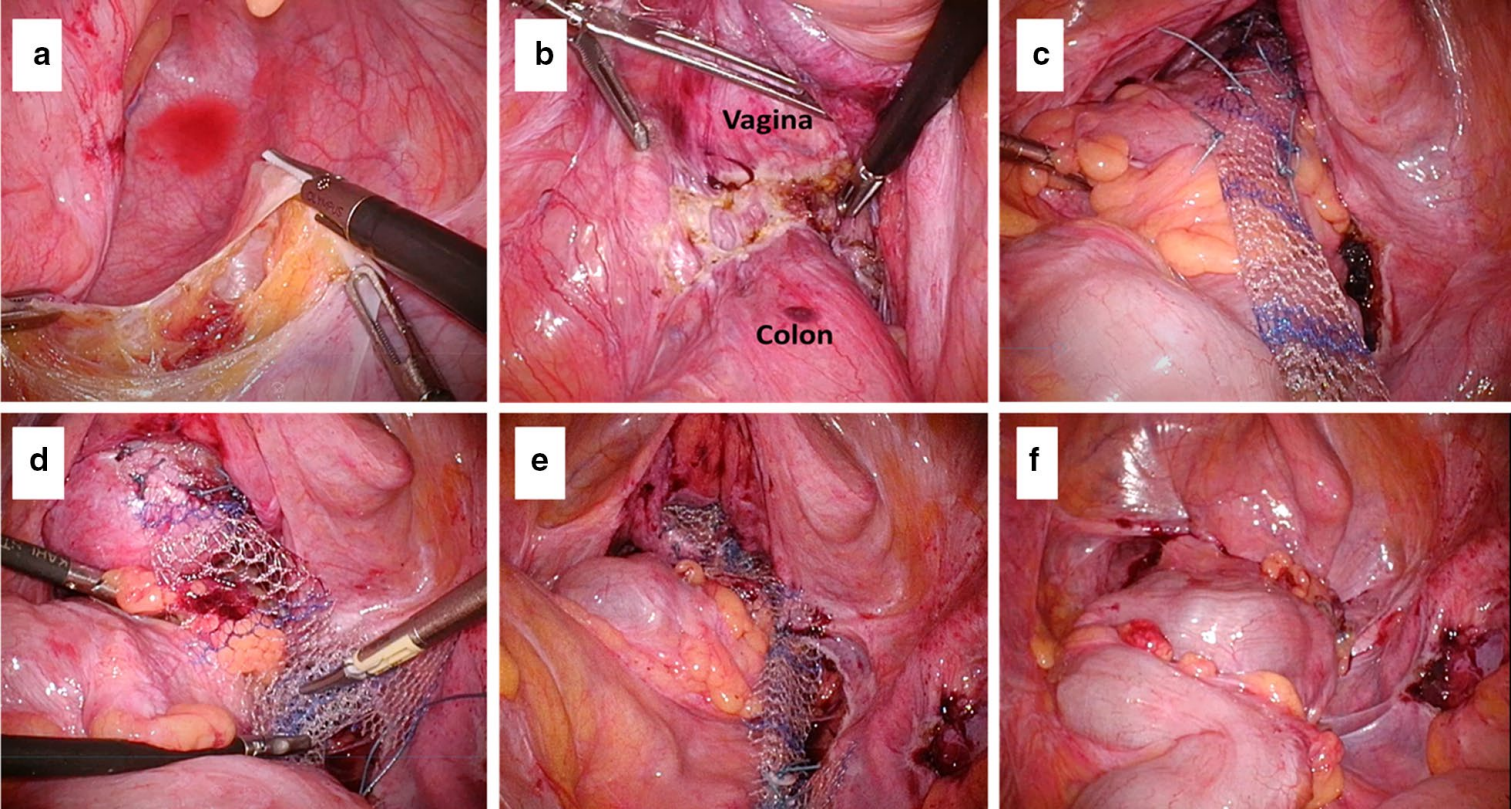
Intra-operative findings. **a** The peritoneum was dissected from the right pelvic wall. Scar and a suture on previous laparoscopic posterior mesh rectopexy were observed (arrow). **b** The rectovaginal space was destroyed in the previous surgery. **c** The self-cut mesh was fixed with non-absorbable suture on the large colon. **d** The self-cut mesh was fixed at the bottom of vesicovaginal space and the top of uterus. **e** The meshes were bridged between the fixed points and the sacral promontory. **f** The meshes were covered with the peritoneum

**Fig. 4:**
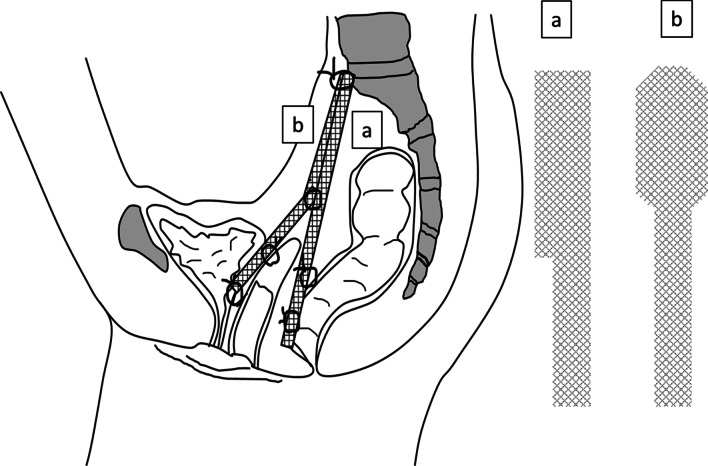
The scheme of surgery. Mesh (**a**) and mesh (**b**) were used at rectovaginal space and vesicovaginal space, respectively

**Fig. 5:**
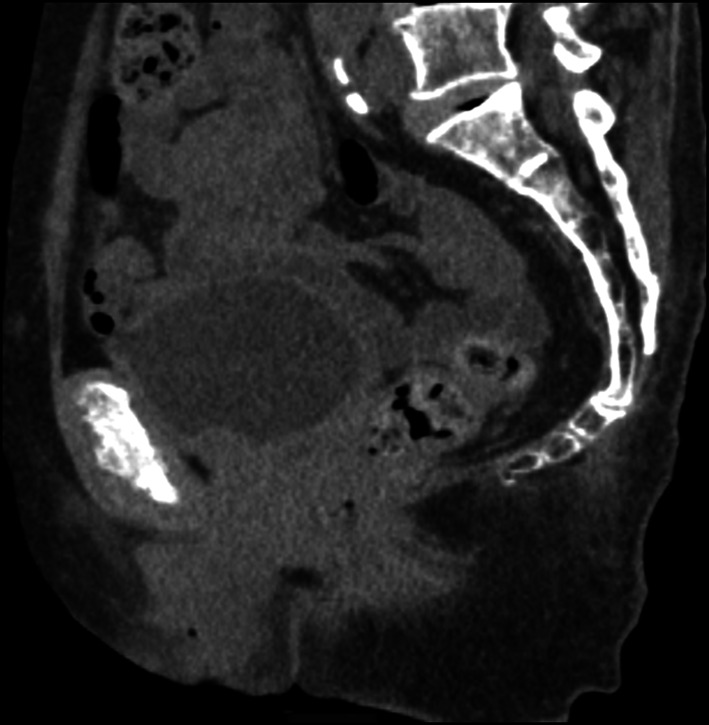
Computed tomography (CT) scan shows no evidence of recurrent rectal prolapse one year later after surgery

We considered about the peritoneal adhesion because we had to cover the mesh with the peritoneum at the end of the surgery. The first port was placed by umbilical incision. As there was no specific adhesion around the pelvic space, we added the four ports and performed the procedure as planned. At first, we dissected the peritoneum on the right pelvic wall from the sacral promontorium to the bottom of pelvic as much as possible avoiding the scar on previous laparoscopic posterior mesh rectopexy (Fig. 3a). As the rectovaginal space was scarring by the influence of the perineal recto-sigmoidectomy, it was not necessary to dissect the rectovaginal space (Fig. 3b). Secondarily, the vesicovaginal space was dissected to the dorsal end of the bladder trigone. Two modified polypropylene meshes (Gynemesh®PS) were fixed with 2-0 non-absorbable sutures (Ethibond Exel®Polyester Suture) at the most distal part of the colon, the dorsal end of bladder and the anterior vaginal wall and then bridged to the longitudinal vertebral ligament on the sacral promontory (Fig. 3c, d). After we combined the two meshes at the middle point, we covered the meshes with peritoneum using absorbable sutures (Fig. 3e, f). The meshes were placed as in the scheme (Fig. 4). The postoperative course was uneventful. One year later, a follow-up computed tomography (CT) scan showed no evidence of recurrence of the rectal prolapse. Pelvic organs settled at their normal positions (Fig. 5). While the symptom of urinary incontinence and rectal prolapse was recovered after the last surgery, the fecal incontinence was not improved so much after each of three surgeries.

## Discussion

While the recurrence rate of external prolapse after the perineal procedure was reported over 20%, a systematic review assessed the outcome of LVMR for external rectal prolapse described that the recurrence rate was 2.8% [1]. As for the reason for the recurrence in our case, we suspected that the durability of the mesh on the second surgery was not enough to pull the full-thickness rectal prolapse.

According to a review from 70 English literatures, LVMR may have better outcome than the perineal procedure for external rectal prolapse [7]. On the other hand, the optimal procedure for recurrent rectal prolapse is still unclear. Although Steele et al. indicated that an abdominal repair had lower recurrence rate than a perineal approach, there are no available review to suggest the optimal procedure for recurrent rectal prolapse due to the variety of surgical techniques [8]. We selected LVMR since our patient had underwent both perineal recto-sigmoidectomy and laparoscopic mesh posterior rectopexy. When we perform LVMR for the primary external rectal prolapse, we normally dissect the rectovaginal space to place the mesh. We were not sure what the rectovaginal space was like since the area had been destroyed in the previous perineal recto-sigmoidectomy. Interestingly, the disappearance of the rectovaginal space made the approach to the most distal of the colon easy. As a result, we were able to complete LVMR smoothly even after the perineal recto-sigmoidectomy.

POP highly occurs in the elderly female and is defined by dropping of pelvic organs from their normal positions. Delancey suggested that POP could be caused by the combination the injury to levator ani muscles and the failure of the connections between the pelvic organs to the pelvic sidewall [9]. As for the treatment for POP, LSC is well-known as one of the popular procedures with high satisfaction and effectiveness [3]. POP is sometimes concomitant with rectal prolapse by a common cause [4, 5]. Nowadays, laparoscopic procedures for the rectal prolapse and POP simultaneously have been also reported with good outcome [10–12]. However, to the best of our knowledge, this is the first report of LVMR and LSC for the recurrent rectal prolapse with POP following the perineal recto-sigmoidectomy and laparoscopic posterior mesh rectopexy.

## Conclusion

We believe that our experience is useful for the physicians who consider LVMR or LSC for recurrent rectal prolapse with POP. On the other hand, we suggest to the physicians to consider the risk of surgical site infection in LVMR compared to the other procedures without mesh for the patient who has the risk of bowel injury when dissecting adhesions caused by the previous surgery.

## Data Availability

Not applicable.

## Abbreviations

POP: Poly-organ prolapse
LVMR: Laparoscopic ventral mesh rectopexy
LSC: Laparoscopic sacrocolpopexy
MRI: The magnetic resonance image
CT: Computed tomography
